# Preventing metastatic emergence of breast cancer

**DOI:** 10.18632/aging.203631

**Published:** 2021-10-11

**Authors:** Alan Wells, Colin Beckwitt, Juan Luis Gomez Marti

**Affiliations:** 1 Department of Pathology, Pittsburgh VA Medical Center and University of Pittsburgh, Pittsburgh, PA 15213, USA

**Keywords:** statin therapy, breast cancer, metastasis, recurrence, secondary prevention

As women age, many are beset by the possibility of recurrence of breast cancers that were found and surgically removed years earlier with no evidence of spread. These cancers can re-emerge as lethal metastases even decades after the initial tumor appears to have been curatively resected, as the breast cancer cells can escape the local tumor, seed distant organs, and become chemo-resistant even before being detected, making targeting of dissemination impractical [1]. To prolong survival, metastatic breast cancers require intensive and debilitating therapies that are poorly tolerated in the elderly [2]. Thus, well-tolerated regimens are desirable to forestall or even prevent these recurrent breast cancers.

Population-based studies have reported a correlation between prolonged progression free interval and overall survival with the use of statins for other health indications [3]. As statins are generally well tolerated, this raises the possibility of using these commonly prescribed agents to avoid recurrence of breast cancer if a number of issues can be resolved. First, the correlation should be of such to allow for secondary prevention usage so as to not overmedicate a population. Second, the mechanism of action should allow for tolerable dosing of the statins in the targeted patients. These two caveats have been addressed. However, lastly, the use of statins should not interfere with treatments should the breast cancer break through the secondary prevention and recur clinically. Fortunately, a recent report completes the picture and answers the third point in the affirmative [4].

A nationwide review of women with breast cancer showed that while the incidence of primary breast cancer was not different in those taking statins, the rate of recurrences was decreased by about a quarter [3]. These data were consistent with studies in other populations that found no difference in incidence, but a similar 20-30% decrease in the rate of metastatic or contralateral breast cancer after a first primary breast cancer is removed [5,6]. These findings supported examining statins further.

The mechanism by which statins achieve the reduction in metastatic disease is still uncertain. The question of whether the effect is directly on the tumor cells or indirectly by reducing cholesterol and fat availability for the cancer cells appears to be settling on the side of direct effects, as the reduction in metastatic incidence is found only with lipophilic statins [3]; as breast cancer cells lack the statin transporters, hydrophilic statins would not impact these carcinoma cells [7] (this correlation does not rule out effects on immune cell functioning that would also be limited to lipophilic statins). As for the direct effects, there are numerous reports of direct cytotoxic effects of statins on carcinoma cells, but cell killing is only achieved at levels of statins in excess of those reached for management of cholesterol levels. Rather, we favor non-cytotoxic effects of statins on carcinoma cells. Through these direct effects, statins act in part to reduce the ability of carcinoma cells to prenylate critical intermediary signaling molecules that are essential to the ability of the quiescent disseminated cells to undergo a transition to metastatic emergence. Our earlier study [8] found that in animal models of spontaneous metastasis and emergence, statin therapy retained the metastatic micrometastases as dormant epithelial carcinoma cells and limited the number of cells that could be converted to emergent and proliferative mesenchymal carcinoma cells. This was found at levels consistent with moderate level therapy, which is well tolerated in most persons.

The last piece of the puzzle related to whether such secondary prevention would limit subsequent cancer therapy should the metastases breakthrough and emerge. Our recent publication [4] addressed that question through use of experimental models of treatment for spontaneous metastases. As the statins keep a large fraction of the disseminated breast cancer cells in the dormant epithelial state, it was possible that this would counteract the cytotoxic effects of chemotherapy. Fortunately, we found that statin pre-therapy and even concurrent therapy do not decrease the ability of two commonly used chemotherapies to kill emergent metastases; if anything, statins enhanced the killing.

Thus, the three criteria for using lipophilic statins for secondary prevention of breast cancer can be supported by the literature. Importantly, the real-world use of such was queried in our recent paper by examining the medical records of 1749 breast cancer patients over a twenty-year period [4]. It was found that in both ER+ and HER2- subsets of breast cancer, statin use was found to reduce recurrence, while the time from recurrence to death was not affected by statins, in line with the mechanism of action being to retain tumor cells in the dormant state. The use of statins in these two groups of mainly women, forestalled recurrence and death by about two years (based on the 50%). This is a significant benefit in both quality of life and overall survival as the gain is due to the prolongation of the clinical silent dormant phase of breast cancer dissemination. Further, this was achieved with use of statins for other indications, and it is likely that the statins were not used for the entire course of the disease. Thus, we propose that these well-tolerated lipophilic statins be considered as secondary preventive agents for women who have had primary breast cancers removed without evidence of clinical metastases to hinder this mortal stage of emergence and recurrence.
